# Neuropathological findings processed by artificial neural networks (ANNs) can perfectly distinguish Alzheimer's patients from controls in the Nun Study

**DOI:** 10.1186/1471-2377-7-15

**Published:** 2007-06-21

**Authors:** Enzo Grossi, Massimo P Buscema, David Snowdon, Piero Antuono

**Affiliations:** 1 Bracco SpA Medical Department, Milan, Italy; 2 Semeion Research Center Sciences of Communication, Rome, Italy; 3 Sanders Brown Center on Aging and Department of Neurology, University of Kentucky, Lexington, Kentucky, USA; 4 Department of Neurology, Medical College of Wisconsin, Milwaukee, USA

## Abstract

**Background:**

Many reports have described that there are fewer differences in AD brain neuropathologic lesions between AD patients and control subjects aged 80 years and older, as compared with the considerable differences between younger persons with AD and controls. In fact some investigators have suggested that since neurofibrillary tangles (NFT) can be identified in the brains of non-demented elderly subjects they should be considered as a consequence of the aging process. At present, there are no universally accepted neuropathological criteria which can mathematically differentiate AD from healthy brain in the oldest old.

The aim of this study is to discover the hidden and non-linear associations among AD pathognomonic brain lesions and the clinical diagnosis of AD in participants in the Nun Study through Artificial Neural Networks (ANNs) analysis

**Methods:**

The analyses were based on 26 clinically- and pathologically-confirmed AD cases and 36 controls who had normal cognitive function. The inputs used for the analyses were just NFT and neuritic plaques counts in neocortex and hippocampus, for which, despite substantial differences in mean lesions counts between AD cases and controls, there was a substantial overlap in the range of lesion counts.

**Results:**

By taking into account the above four neuropathological features, the overall predictive capability of ANNs in sorting out AD cases from normal controls reached 100%. The corresponding accuracy obtained with Linear Discriminant Analysis was 92.30%. These results were consistently obtained in ten independent experiments. The same experiments were carried out with ANNs on a subgroup of 13 non severe AD patients and on the same 36 controls. The results obtained in terms of prediction accuracy with ANNs were exactly the same.

Input relevance analysis confirmed the relative dominance of NFT in neocortex in discriminating between AD patients and controls and indicated the lesser importance played by NP in the hippocampus.

**Conclusion:**

The results of this study suggest that: a) cortical NFT represent the key variable in AD neuropathology; b) the neuropathologic profile of AD subjects is complex, however, c) ANNs can analyze neuropathologic features and differentiate AD cases from controls.

## Background

Both Neurofibrillary tangles (NFT) and neuritic plaques (NP) are the primary neuropathologic markers of Alzheimer's disease (AD), although they are highly prevalent in normal brain aging [1-4].

Many reports have described that there are fewer differences in AD brain neuropathologic lesions between AD patients and control subjects aged 80 years and older, as compared with the considerable differences between younger persons with AD and controls [5,6]. While there are dramatic differences in neuropathologic lesion counts between middle-aged AD cases and controls, the difference in lesion counts, while significant, is of lesser magnitude in older adult AD cases and controls[5].

Advanced age at death is associated with somewhat less severe dementia and fewer senile plaques and neurofibrillary tangles[6].

Presently there is not a consensus on whether NFT constitute a specific effect of the disease or result, in part, from a non-specific age related process.

In fact, some investigators [7] have suggested that, since the NFT are very prevalent in the brains of non-demented older adults, the presence of NFT in the brain is not, by itself, diagnostic of AD, and that NFT should be viewed as a later occurrence in the pathological progression of the disease.

Overall, the exact role of NFT to AD, aging, and dementia remains unclear. Even universally accepted neuropathological criteria for Alzheimer's disease differ on the diagnostic role of NFT.

The current approach of determining different cut-off points for NFT and NP density and regional distribution do not allow a 100% sensitivity and specificity in discriminating between AD brains and control subjects with normal cognitive function.

Recent studies further suggest that NFT have a stronger correlation to cognitive function than NP, not only in AD but also in normal aging and mild cognitive impairment [1,3,8]. The degree of cognitive impairment is a function of the distribution of NTF within the brain [7]. In particular, the presence of high NFT density in the entorhinal and hippocampus neurons is strongly correlated to reduced cognitive performance in normal aging, whereas NFT formation in neocortical areas is associated with clinically overt AD [2-4,9].

Neuropathologic studies [2-4,9] have shown that the distribution of NFT in the human brain follows, in general, a predictable and hierarchical pattern whereas the distribution of NP varies among individuals. Neurofibrillary pathology is initially limited to the hippocampus and the entorhinal cortex [3,9]. As the number of NFT increases in these areas, neurofibrillary pathology extends into the temporal cortex. Finally, tangles emerge and spread to the neocortical areas of the brain.

In a previous study [10] we have shown that Artificial Neural Networks analysis applied to demographic, clinical and genotype descriptors allowed a better prediction of the number of NFT in the neocortex and hippocampus than the number of NP in the same areas. These results indicate that a non-linear analysis of complex data is a valid approach in highlighting on the role of NP and NFT in the development of a degenerative process leading to AD. This supports the concept that the presence of NFT in aging may represent one of its earliest pathological substrates and play a significant role in the initial stages of memory impairment, confirming the findings [3,9] by other authors.

An important way to challenge this hypothesis is to evaluate the predictive role of NFT and NP in two critical brain regions, i.e. neocortex and hippocampus, in distinguishing between normal subjects and those with AD.

The aim of this study is to discover the hidden and non-linear associations among Alzheimer's disease pathognomonic brain lesions and the clinical diagnosis of Alzheimer's disease in participants in the Nun Study.

## Methods

### Subjects

Subjects in the study were selected from a cohort of 117 participants in the Nun Study who had donated their brains [10]. The Nun Study was approved by the University of Kentucky's Institutional Review Board. In order to select control subjects with normal cognitive function we excluded non-demented subjects with a MMSE score equal or less than 24 and/or the concomitant presence of mild cognitive impairment of the amnesic type [11].

Thirty six subjects matched these criteria. Six of them were ApoE4 positive (16.6%).

Selection criteria for pure AD patients was the presence of clinical dementia and values of NFT and NP in the neocortex and hippocampus above the following cut-off:

*Neurofibrillary Tangles in Neocortex*: average value of neocortical NFT per mm^2 ^> 1.0;

*Neurofibrillary Tangles in Hippocampus*: average value of hippocampal NFT per mm^2 ^> 10;

*Neuritic Plaques in Neocortex*: maximum number of NP in the neocortex >1.5;

*Neuritic Plaques in Hippocampus*: maximum number of NP in the hippocampus >1.5.

These cut-off derive from a previous mathematical validation of neuropathological values distribution observed in a previous study [10].

Twenty six patients fulfilled these criteria and they constitute the AD cases in this analyses. Nine of them were ApoE4 positive (34.6%).

### Artificial neural networks analysis

#### ANNs structure and architecture

ANNs models were constructed by using non commercial programs developed by Semeion Research Center [12-17]. In this experiment several ANN architectures with different learning rules were assessed, all of them sharing the following structure: the input vector had number of nodes equal to the number of independent variables, the output vector had two nodes corresponding to the two different outcomes (AD cases vs normal controls), and a single layer of hidden units

ANNs with Back Propagation learning rule were employed sharing the following structure: the input layer had a number of nodes equal to the number of independent variables, the output layer had two nodes corresponding to the target (AD cases/normal controls), and the inner layer had four hidden units.

Results obtained with those neural networks have been compared with a linear statistical model: the Linear Discriminant Analysis (LDA) (Software SPSS^®^) using the same training and testing subsets.

During the training phase the input relevance of each variable was assessed. The so called "input relevance" is a parameter expressing the magnitude of the activation of a given node during the training phase. The magnitude of the activation is arbitrarily expressed with a number which ranges from zero to infinity.

In technical terms, the "Input Relevance" is the Fan-out of every input when the ANN is trained:

Ri=1K⋅∑cK∑jNwc,j,i;
 MathType@MTEF@5@5@+=feaafiart1ev1aqatCvAUfKttLearuWrP9MDH5MBPbIqV92AaeXatLxBI9gBaebbnrfifHhDYfgasaacH8akY=wiFfYdH8Gipec8Eeeu0xXdbba9frFj0=OqFfea0dXdd9vqai=hGuQ8kuc9pgc9s8qqaq=dirpe0xb9q8qiLsFr0=vr0=vr0dc8meaabaqaciaacaGaaeqabaqabeGadaaakeaacqWGsbGudaWgaaWcbaGaemyAaKgabeaakiabg2da9maalaaabaGaeGymaedabaGaem4saSeaaiabgwSixpaaqahabaWaaabCaeaacqWG3bWDdaWgaaWcbaGaem4yamMaeiilaWIaemOAaOMaeiilaWIaemyAaKgabeaaaeaacqWGQbGAaeaacqWGobGta0GaeyyeIuoaaSqaaiabdogaJbqaaiabdUealbqdcqGHris5aOGaei4oaSdaaa@46B5@

*where*:

*R*_*i *_is the mean relevance of the i-th input variable of the dataset;

K is the number of classifiers used in the training phase;

*N *is the number of hidden units of the K classifiers trained;

*w*_*c*,*j*,*i *_is the trained weight of the c-th classifier, connecting the i-th input to the j-th hidden unit.

#### The Validation Protocol

The validation protocol is a fundamental procedure to verify the models' ability to generalize the results reached in the Testing phase of each model. The application of a fixed protocol measures the level of performance that a model can produce on data that are not present in the Testing and/or Training sample. Different types of protocol exist in the literature, each presenting advantages and disadvantages.

The protocol, from the point of view of a general procedure, consists of the following steps:

1. subdividing the database in a random way into two subsamples: Subsets A and B;

2. train an ANN on Subset A; in this phase the ANN learns to associate the input variables with those that are indicated as targets;

3. at the end of the training phase the weight matrix produced by the ANN is saved and frozen together with all the other parameters used for the training;

4. with the weight matrix saved, Subset B, which it has not seen before, is shown to the ANN, so that in each case the ANN can express an evaluation based on the previous training; this operation takes place for each input vector and every result (output vector) and is not communicated to the ANN; the ANN is in this way evaluated only in reference to the generalization ability that it has acquired during the Training phase;

5. a new ANN is constructed with identical architecture to the previous one and the procedure is repeated from point 1; but this time the ANN will be trained on Subset B and blindly tested on the Subset A.

This general training plan has been further articulated with the aim of increasing the level of reliability in terms of generalization of the processing models. More specifically we employed the so-called 5·2 cross-validation protocol [13]. In this procedure the study sample is randomly divided ten times into two sub samples, always different but containing a similar distribution of cases and controls: the training one (containing the dependent variable) and the testing one. During the training phase the ANN learns a model of data distribution and then, on the basis of such a model, classifies subjects in the testing set in a blind way. The training and testing sets are then reversed and consequently 10 analyses for every model employed are conducted. To compare the ANNs performances, the same protocol was used with the same data distribution to validate the Linear Discriminant Analysis (LDA).

## Results

Table 1 shows the descriptive variables of the subjects included in this study according to the above criteria.

**Table 1:** Characteristics of the sample under evaluation

	**AD (n = 26)**				**Normal (n = 36)**			
	
**Feature**		**Range**				**Range**		
						
	**mean**	**min**	**max**	**SD**	**mean**	**min**	**max**	**SD**
**Age at last exam**	89.73	79.27	100.65	5.07	83.72	76.24	101.09	6.07
**Education years**	14.85	8.00	18.00	3.31	16.44	8.00	18.00	2.25
**ADL**	1.73	0.00	5.00	2.09	4.61	0.00	5.00	1.15
**WRCL**	0.23	0.00	2.00	0.59	6.58	4.00	9.00	1.27
**CNPR**	4.19	0.00	11.00	4.36	10.11	5.00	11.00	1.24
**BOST**	4.31	0.00	14.00	4.60	12.42	0.00	15.00	2.76
**VRBF**	2.96	0.00	14.00	3.94	14.00	8.00	23.00	3.91
**MMSE**	7.62	0.00	23.00	8.74	27.83	25.00	30.00	1.36
**Mean NFT neocortex**	22.03	1.47	61.99	16.85	0.29	0.00	4.88	0.83
**Mean NFT Hippocampus**	48.53	12.80	94.90	23.71	9.36	0.00	59.73	15.41
**MaxNP neocortex**	10.79	3.83	21.28	4.31	3.13	0.00	11.06	3.43
**Max NP Hippocampus**	6.02	1.70	13.62	3.66	1.45	0.00	15.74	3.09

WRCL: Delayed Word Recall score; CNPR: Constructional Praxis score; BOST : Boston Naming score; VRBF: Verbal Fluency score; MMSE: Mini-Mental State Examination

As one can see, even if the average difference between the neuropathological lesion load in the two groups was substantial, a marked overlap of values was present for NFT in hippocampus, NP in neocortex, and NP in hippocampus.

A good linear relationship between each of the 4 selected input variables and the target of the study (AD cases/normal controls) was present: for *Neurofibrillary Tangles in Neocortex*, r-squared = 0.50; *Neurofibrillary Tangles in Hippocampus*, r-squared = 0.50; *Neuritic Plaques in Neocortex*, r-squared = 0.50; *Neuritic Plaques in Hippocampus *respectively. r-squared = 0.32 ;

By taking into account all the four recorded neuropathological features, the overall predictive capability of ANNs in sorting out AD from normal amounted consistently to 100% (table 2).

**Table 2:** Performance of the ANNs in discriminating AD cases from normal controls. The analysis was carried out on all 4 neuropathologic variables registered in the original database of patients in ten separated experiments with different training and testing subsets. Linear Discriminant Analysis [LDA] results on the same subsets are shown for comparison.

Tr and Ts subsets	ANN			LDA		
	AD	Normal	Mean accuracy	AD	Normal	Mean accuracy

FF_Bp*(4 × 2)1a	100.00%	100.00%	100.00%	100.00%	87.50%	93.75%
FF_Bp(4 × 2)1b	100.00%	100.00%	100.00%	100.00%	91.67%	95.83%
FF_Bp(4 × 2)2a	100.00%	100.00%	100.00%	100.00%	72.73%	86.36%
FF_Bp(4 × 2)2b	100.00%	100.00%	100.00%	100.00%	88.89%	94.44%
FF_Bp(4 × 2)3a	100.00%	100.00%	100.00%	100.00%	87.50%	93.75%
FF_Bp(4 × 2)3b	100.00%	100.00%	100.00%	100.00%	83.33%	91.67%
FF_Bp(4 × 2)4a	100.00%	100.00%	100.00%	100.00%	72.73%	86.36%
FF_Bp(4 × 2)4b	100.00%	100.00%	100.00%	95.00%	100.00%	97.50%
FF_Bp(4 × 2)5a	100.00%	100.00%	100.00%	100.00%	91.67%	95.83%
FF_Bp(4 × 2)5b	100.00%	100.00%	100.00%	100.00%	75.00%	87.50%

**Average**	**100.00%**	**100.00%**	**100.00%**	**99.50%**	**85.10%**	**92.30%**

* Feed Forward Back Propagation Neural Network

Tr: Training set; TS: Testing set

These results were consistently obtained in ten separated experiments performed on different training and testing subsets. The corresponding results obtained with LDA were good but not excellent; in fact the mean accuracy rate was 92.30%.

Since some AD patients had severe cognitive impairment, in further experiments, we excluded from the analysis AD patients with MMSE score below 4.

A subset of 13 AD patients was obtained with a mean MMSE equal to 15.

The average values of pathological markers didn't differ between these two subgroups with the exception of NFT in neocortex (Table 3). We repeated the same predictive experiments on a new data set composed of these 13 mild AD patients and the same 36 controls obtaining identical results.

**Table 3:** Comparison between severe and non severe AD patients.

**Variables**	**Mean**	**SD**	**Mean**	**SD**	**p value**
Age at last exam.	91.63	5.88	88.69	4.14	n.s.
Education years	15.69	2.81	14.00	3.65	n.s.
ADL	3.23	1.88	0.23	0.83	<0.001
WRCL	0.46	0.78	0.00	0.00	<0.001
CNPR	7.85	2.64	0.54	1.94	<0.001
BOSTON	8.31	2.95	0.31	0.85	<0.001
VRBF	5.92	3.66	0.00	0.00	<0.001
MMSE	15.00	6.36	0.23	0.60	<0.001
Mean NFT neocortex	29.31	17.24	14.74	13.39	<0.001
Mean NFT Hippocampus	50.31	22.99	46.75	25.22	n.s.
Max NP neocortex	11.65	4.81	9.92	3.73	n.s.
Max NP Hippocampus	5.34	4.12	6.71	3.15	n.s.

In order to assess the relative importance of the four neuropathological AD markers in developing the model build by ANNs, in the ten experiments we evaluated the so called "input relevance " of each markers during the training phase of the neural network.

**Figure 1:**
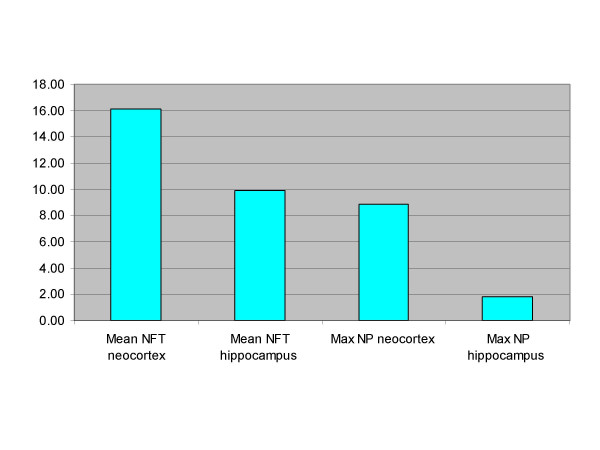
Mean input relevance* of neuropathological markers in ANNs experiments. * Input relevance refer to the ranking of each variable in term of relative importance within the model created by artificial neural networks. The higher the value, the higher the importance of the variable.

Figure 1 shows the average input relevance of each variable in the ten independent training sessions. As one can see, NFT Neocortex accounted for the highest input relevance followed by NFT Hippocampus, NP Neocortex, and lastly by Max NP Hippocampus.

## Discussion

Artificial neural networks have shown optimal performance on various medical applications because of their capacity to learn how to identify complex relationships among data.

At variance with statistical linear methods, ANNs are able to reproduce the dynamic interaction of multiple factors simultaneously, allowing the study of complexity; they can also draw conclusions on an individual basis and not as average trends.

In a previous paper [10] we have shown that ANNs can be used to predict the results of post-mortem brain evaluations from cognitive performance data among 117 participants in the Nun Study.

That is, we determined how demographic data and cognitive and functional variables of each subject during the last year of her life could predict: a) the presence of brain pathology expressed as Braak stages of AD pathology, NFT and NP count in the neocortex and hippocampus; and b) brain atrophy, a highly prevalent neuropathologic feature of AD.

In this study our goal was to understand what constitutes the relevant neuropathological pattern differentiating AD from normal control subjects, an issue which, so far, has never been solved.

Thanks to the ANNs analysis we succeeded in reaching a perfect distinction between the two groups which remained unchanged even when we analyzed only the clinically mild and moderate AD patients. Input relevance analysis confirmed the relative dominance of NFT in the neocortex in discriminating between normal controls and AD cases and indicated the low importance played by NP in hippocampus.

Input relevance is a practical way to open the so called "black box" of ANNs, allowing one to discover the role played by each variable in the developing the data model during the training phase. The numerical value of this parameter is proportionally related to the "weight" of a given variable in the model.

Another major challenge in comparing the prevalence of AD lesions in old individuals with AD and non-demented control subjects is the selection of appropriate criteria for excluding mild dementia in the controls. In fact, as regards to non-demented people most of the studies rely on the interview of a knowledgeable informant after the subject death, rather than direct observation of the control subject, according to the same protocol used to assess AD patients One example is the study published by Berg and co-workers in 1998 [5], in which experienced nurses or physicians interviewed informants and reviewed the records of previous clinical assessments to define the Clinical Dementia Rating score of controls. In addition, some controls were excluded because of neocortical senile plaques densities that met neuropathological criteria for AD, introducing in this way a circular reasoning.

A possible limitation of our analysis is linked to the relative small sample size. This issue can be considered at two different levels: the statistical and epidemiological one.

From a pure statistical point of view we can say that the small number of variables considered guarantees a balanced ratio between variables and records. In addition the use of a rigorous validation protocol with many training and testing procedures should protect against statistical imbalances.

From an epidemiological point of view we can't regard the 26 patients in this study as a representative population of AD patients. Therefore it is clear that the results presented in the paper are only valid for this particular environment and cannot be generalized. One should anyway consider the extreme scarcity in the general literature of autopsy data in groups of aged people with a substantial proportion of individuals without dementia symptoms.

Another potential limitation of our paper is that the markers that might best correlate with cognitive status (i.e. synaptic markers) are not included in the dataset ; nonetheless, we think that the information carried out by NFT and NP is sufficiently specific to make a considerable contribution to the understanding of pathology-clinical relation.

## Conclusion

In conclusion, the results of this study confirm that the neuropathologic profile of AD subjects is complex but specific and thanks to ANNs it can be conveniently differentiated from that of normal subjects. Cortical NFT represent the key variable more likely related to the patho-physiology of the disease than the NP.

## Competing interests

The author(s) declare that they have no competing interests.

## Authors' contributions

EG conceived the paper and wrote the manuscript

MB performed ANN analyses and participated in the design and coordination of the study.

DS directs the Nun Study and assisted in the writing of the manuscript

PA participated in manuscript finalisation and coordination

All authors read and approved the final manuscript.

## Pre-publication history

The pre-publication history for this paper can be accessed here:


